# Pleiotropic use of Statins as non-lipid-lowering drugs

**DOI:** 10.7150/ijbs.42965

**Published:** 2020-08-13

**Authors:** Qijia Zhang, Jianlong Dong, Ze Yu

**Affiliations:** 1 Digestive internal medicine and Department of infectious diseases, Zhuhai Hospital of Integrated Traditional Chinese and Western Medicine, Zhuhai, China.; 2 College of Life Science, Northeast Agricultural University, Harbin, China.; 3 Affiliated Cancer Hospital and Institute of Guangzhou Medical University, State Key Laboratory of Respiratory Disease, Guangzhou, China.

**Keywords:** Statins, mevalonate pathway, non-lipid-lowering function, anticancer agents

## Abstract

Statins, known as HMG-CoA reductase (HMGCR) inhibitors, have primarily been utilized for metabolic and angiographic medical applications because of their cholesterol-lowering effects. Similar to other drugs, statins may also induce a series of potential side effects. Statins inhibit the HMGCR (rate-limiting enzyme) activity in early stages of mevalonate pathway and then indirectly affect a number of intermediate products, including non-sterol isoprenoids (coenzyme Q10, dolichol etc.), which can result in impaired functions of body organs. Recently, scores of studies have uncovered additional functional mechanisms of statins in other diseases, such as diabetes mellitus, nervous system diseases, coronary heart disease, inflammation and cancers. This review aims to summarize the positive and adverse mechanisms of statin therapy. Statin care should be taken in the treatment of many diseases including cancers. Since the underlying mechanisms are not fully elucidated, future studies should spend more time and efforts on basic research to explore the mechanisms of statins.

## Introduction

Statins are a class of drugs that contain a naphthyl ring skeleton structure and are known as HMGCR inhibitors, which regulate the concentration of plasma lipoproteins by inhibiting cholesterol synthesis. Statins could reduce the synthesis of cholesterol, thus increasing the number and activity of low-density lipoprotein (LDL) receptors on the membrane of liver cells and enhancing the clearance of plasma cholesterol 1, 2. Since the hydrophilic group of statins is similar to the HMG portion of the substrate 3, statins can be used as competitive inhibitors of HMG-CoA. Therefore, statins can reduce cholesterol synthesis to prevent cardiovascular diseases such as atherosclerosis 4-6.

Mevastatin was the first discovered HMG-CoA reductase inhibitor and purified by Akira Endo from the mold fermentation nutrient medium 7. Then, an analogue of lovastatin was isolated from the broth of *Aspergillus terreus* in 1980 and marketed by Merck in 1987. Subsequently, other statin, including lovastatin and simvastatin, were discovered and studied 8, 9. Such natural micro-molecules were recognized as the first generation of HMG-CoA reductase inhibitors, retaining the highly absorptive, fat-soluble and hypo-toxic characteristics 10. However, complicated synthesis processes and expensive costs had restricted their applications. Thus, artificial modifications of statin structure were required to reduce the costs. The subsequent second generation of statins, such as pravastatin and fluvastatin, had higher solubility in water, lower lipid solubility and faster oral absorption than the first generation 9.

As an important component in the electron transport chain during cellular respiration, coenzyme Q10 (CoQ10) (ubiquinone) mediates electron transfer and results in the shifting of hydrogen ions into the mitochondrial intermembrane space to form a hydrogen ion concentration gradient, ultimately required for the formation of ATP 11. Statins, along with inhibiting the activity of HMG-CoA reductase enzyme, also block the pathway leading to the production of CoQ10 12, 13. The lack of CoQ10 resulting from statins may block normal cellular aerobic respiration and produce an abundance of free radicals, which are toxic to cells 12, 14.

## The Diabetogenic mechanisms of Statins and clinical implications

Recently, clinical trials and observational studies have indicated an approximate 10% increase in new-onset diabetes mellitus among patients taking statins, but the mechanisms underlying these clinical effects are not yet fully understood. Clinical and basic studies have shown that statins can potentially increase plasma glucose levels and diabetes risk by enhancing insulin resistance or impairing β-cell function. Here, we describe the potential mechanisms underlying how statins affect glucose homeostasis. Many studies have indicated that the effects of statins in diabetes are related to the inhibition of HMG-CoA reductase activity 15.

Statin-induced cholesterol depletion can decrease GLUT4 gene expression and increase GLUT1 protein levels 16. Moreover, membrane cholesterol depletion results in a remarkable decline in the tyrosine-specific phosphorylation of insulin receptor substrate (IRS) and the serine-threonine phosphorylation of AKT in response to insulin 17. IRS1/2 mediate the control of various cellular processes by insulin. When phosphorylated by the insulin receptor, IRS1/2 bind specifically to various cellular proteins containing SH2 domains. Statins can downregulate GLUT4 gene expression and reduce insulin-stimulated glucose transport in adipocytes, while adding mevalonate can reverse these effects; HMG-CoA reductase catalyzes this process 16. These results indicate that statins regulate the GLUT4 expression via influencing mevalonate pathway and some intermediate metabolites in mevalonate pathway play an important role in regulating transcription levels of GLUT4. Besides, statins also reduce expression level of the small GTP-binding protein RhoA, which is a cell membrane-anchored protein and requires isoprenylation to activate it. Isoprenylation of RhoA is crucial to support cholesterol homeostasis 18. These results suggest that the reduction of isoprenoid intermediates in the cholesterol biosynthetic pathway is related to insulin resistance.

**Figure 1:**
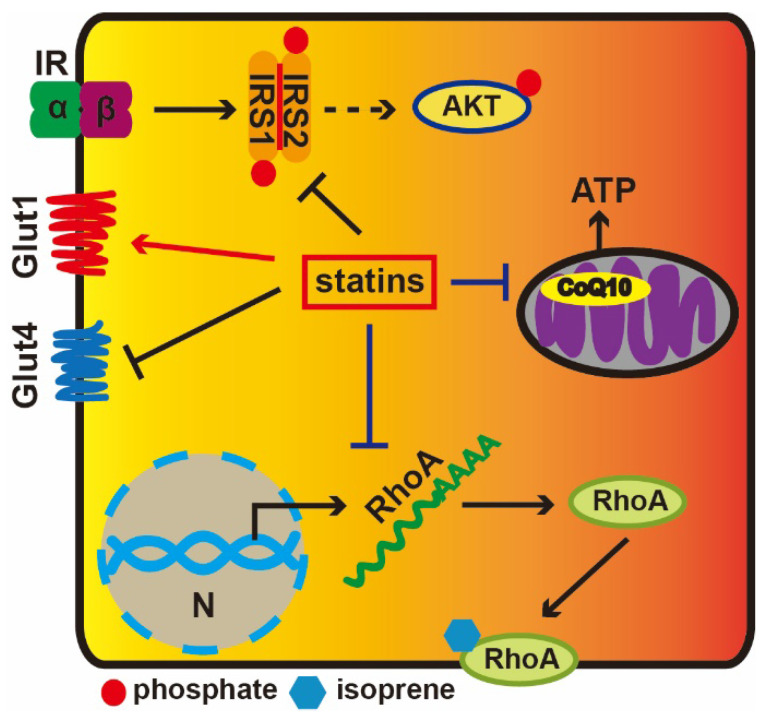
** The diabetogenic mechanisms of statins.** Extracellular insulin can activate Insulin receptor tyrosine kinase, which results in the phosphorylation of IRS1, and AKT is subsequently activated. Statins could facilitate GLUT1 transcription, while repress GLUT4. Statins prevent RhoA from transferring to the cell membrane via inhibiting its translation. Statins reduce CoQ10 levels via depressing the HMG-CoA activity to inhibition of the mevalonate pathway impairing mitochondrial electron transport chain and ATP production.

Isoprene residues are among the important intermediate molecules of the mevalonate synthesis pathway and are also required for the synthesis of CoQ10 19. CoQ10 participates in mitochondrial oxidative phosphorylation and the generation of ATP (**Figure 1**). Both *in vivo* and *in vitro* experiments have proved that simvastatin reduce ATP production in a dose-dependent manner and that insulin secretion requires ATP 20. Depressed coenzyme Q10 levels are likely to impact β-cell function, decrease insulin sensitivity, disturb glucose tolerance and inhibit oxidative phosphorylation in mitochondria 21. Additionally, the accumulation of small G-proteins is paralleled by a reduction in insulin levels. It is known that lovastatin can decrease glucose-induced insulin secretion from pancreatic islets by 50%, while mevalonate can rescue this effect 22.

Our purpose is to explore possible risks underlying the effects of statins in diabetogenic clinical treatment. Relevant risk factors related to diabetes during the administration of statins, such as statin variety, treatment dose and hazards for patients with type II diabetes, are very important to be considered in the treatment of diabetic patients. Some studies have found that patients with diabetes receiving statin therapy appear to suffer more risk factors for type II diabetes, particularly in elderly age (age >70 years) 23.

## Statin therapy for nervous system diseases

Clinically, a great body of evidence indicates statins are employed to treat atherosclerosis in patients with cerebral thrombosis and Alzheimer's disease as it can significantly reduce the incidence of ischemic and hemorrhagic stroke 24. Despite that numerous clinical studies seek to confirm the therapeutic potential of statins in various central nervous system disorders, including multiple sclerosis, epilepsy, dementia, depression and stroke, there still has been a gap in our understanding of mechanisms underlying the neurological effects of statins. In 2006, it was announced that lower cholesterol levels and stroke prevention studies represented the first milestone for the prevention of recurrent stroke in patients administered statins after stroke. These results suggest that despite the simultaneous reduction of cardiovascular events in the statin intervention group, the incidence of hemorrhagic stroke increased slightly, although bleeding in the two groups did not increase the number of deaths caused by stroke, and the incidence of hemorrhagic stroke was extremely low (2.3%; 1.4% in the control group) 25.

**Figure 2:**
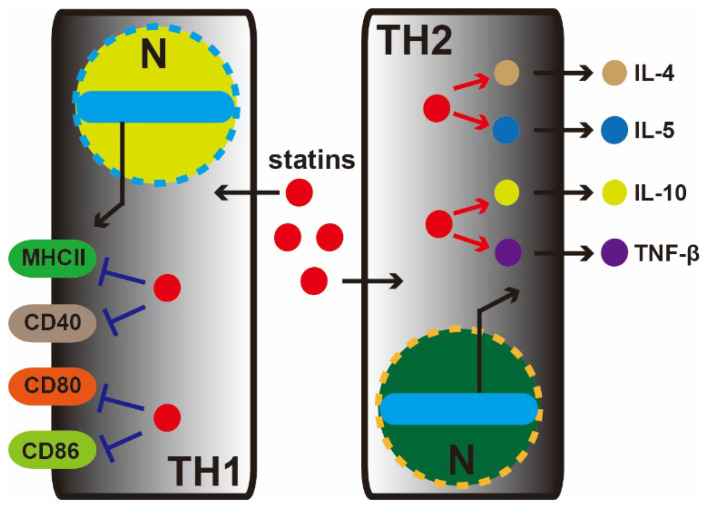
** Statin therapy for nervous system diseases.** In the central nervous system, statins decrease the infiltration of inflammatory cells (TH1 and TH2 cell). In the TH1 cell, statins depress MHC-II, CD40, CD80, and CD86 marker proteins expression. Meanwhile, conversely, in TH2 cell, statins increase the secretion of TH2 cytokines (IL-4, IL-5, IL-10, and TNF-β).

Studies from Yousself et al. have shown that statins decrease the infiltration of the central nervous system (TH1 cell) with inflammatory cells, reduce the expression of major histocompatibility complex II (MHC-II) and inhibit CD40, CD80, and CD86. Oral atorvastatin can prevent and reverse chronic relapses of multiple sclerosis in patients with clinical symptoms of paralysis. Atorvastatin can increase the secretion of TH2 cytokines (IL-4, IL-5, IL-10 and TNF-β) (**Figure 2**). Mevalonate can reverse the effects of atorvastatin in T cells and antigen-presenting cells and inhibit isoprenoid synthesis, thereby preventing intracellular signaling 26.

Recent studies also suggest that statins can mitigate Alzheimer's disease (AD). One study found that among 845 cases of elderly patients (average: 80.5 years old), 20.1% had dementia, while in the 10.9% of people without dementia taking lipid-lowering medication, only 3.5% developed dementia. AD is one of the most common causes of dementia, and taking statins may reduce morbidity, improve cognitive function and delay the progression of AD; thus, statin treatment brings new hope to AD therapy 27.

The association between long-term statin use and adverse cognitive effects has not been well established. A recent review analyzed the MedWatch drug surveillance system and found statins could also cause memory loss in some patients. It was reported that 60 cases of memory loss were associated with statin use, of which 36 cases took simvastatin, 23 cases took atorvastatin, 1 took pravastatin. Memory loss occurred within 2 months after taking statins in 50% of patients. Among 25 cases taking simvastatin, 14 patients developed improved memory after stopping the drug. However, many of the studies evaluating the effects of statins on cognitive dysfunction have a relatively short follow-up, limiting the ability to assess whether there is a true caused relationship between long-term statin use and cognitive function 28. Consequently, we cannot evaluate whether or not the risk of statin-associated cognitive side effects increases with time.

## Statins for the treatment of Coronary Heart Disease

Statins can be used to treat coronary heart disease by alleviating endothelial dysfunction, inhibiting adhesion between leukocytes and endothelial cells, resisting oxidation, stabilizing plaques, affecting blood flow, suppressing smooth muscle cell proliferation and exerting other effects 29, 30. Research has shown that statins affect vascular smooth muscle cell proliferation that is related to Rho protein isoprenylation. Rho protein not only plays a key role in maintaining cell morphology but also is responsible for degrading cell cycle inhibitory p27 31. Additionally, statins could reduce the number of Rho proteins attached to cell membrane and decrease Cdk2 expression level in vascular smooth muscle cells, ultimately inhibiting cells from transitioning between G1 to S phase and causing cell cycle arrest 32.

To study whether statins can suppress cell proliferation via affecting DNA replication in rat aorta vascular smooth muscle cells, Bruemmer et al. selected two chromosomal proteins, MCM6 and MCM7, which played important roles in DNA replication initiation stage. Results showed that MCM6 and MCM7 expression was suppressed by atorvastatin in a dose-dependent manner, and mevalonate could completely reverse the effect. And their study also found that atorvastatin may inhibit MCM6 and MCM7 enhancer activity. Meanwhile, since the MCM6/7 enhancer contains several E2F transcription sites, adenovirus-mediated overexpression of E2F could reverse the inhibition exerted on MCM6 and MCM7 by atorvastatin. These results suggested that atorvastatin was associated with MCM6 and MCM7 mRNA transcription 33.

**Figure 3:**
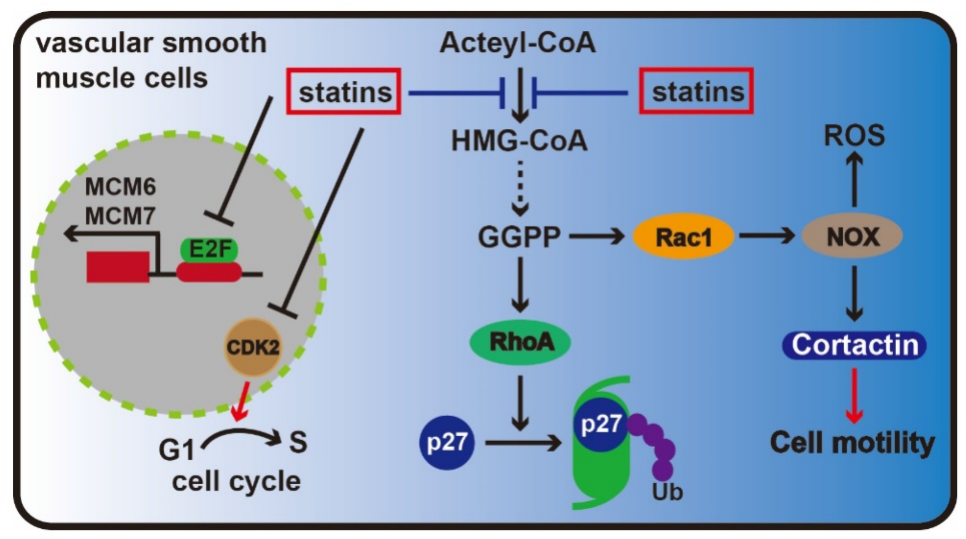
** Statin therapy for coronary heart disease.** In the vascular epithelial cells, the key effectors are Rac1 and RhoA protein in the mevalonate pathway, Rac1 can maintain the stability of the cytoskeleton and promote cell migration via regulating NOX, and RhoA protein can keep from the ubiquitin degradation of p27, inhibit cell cycle progression. As the inhibitor of the mevalonate pathway, statins are able to inhibiting the formation of Rac1 and RhoA. Meanwhile, statins can also prevent the transition of G1-S phase through inhibiting CDK2. Moreover, statins inhibit cell proliferation by depressing the transcription of MCM6 and MCM7.

Recent advances have shown that Rac1 GTPase is a major master regulator of cell motility through regulating the generation of reactive oxygen species and cortical actin reorganization, which are induced by regulating nicotinamide adenine dinucleotide phosphate (NADPH) oxidase activity 34. Statins can inhibit Rac1 activity (**Figure 3**). One critical issue is to evaluate the activation of Rac1 GTPase, which is a key component of cardiovascular pathologies, including cardiac hypertrophy, fibrosis and atrial fibrillation 35. However, the signal transduction involved in these processes has yet to be deciphered. An improved understanding of the Rac1-mediated effects of statins may help us to identify novel therapeutic strategies and targets.

## The anti-inflammatory effects of Statins

Basic and clinical studies have demonstrated statins' anti-inflammatory effects 36, 37. The primary mechanism involves in cell proliferation inhibition, aggregation of inflammatory cells, and the secretion of cellular factors, increasing endothelial nitric oxide production, and protecting endothelial function 38.

Nuclear factor κB (NF-κB) is an important transcription regulatory protein and it is a homo- or heterodimeric complex formed by the Rel-like domain-containing proteins. It is involved in activation of immune cells, T and B lymphocyte development, the stress response, apoptosis and other cellular activities 39. Its abnormal activation can exacerbate inflammatory reactions. Chemokines, adhesion molecules and other inflammation-related gene promoters all contain nuclear factor NF-κB recognition sites. Statins not only inhibit the activity of nuclear factor κB expression, but also prevent NF-κB from entering the nucleus, and ultimately reduce the expression of adhesion molecules and inflammatory factors 40.

**Figure 4:**
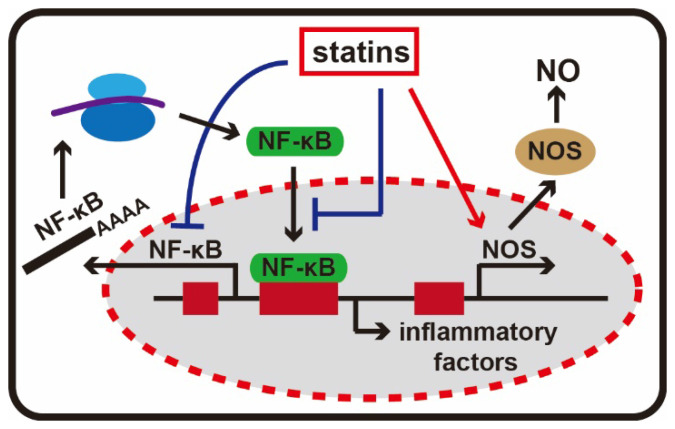
** The anti-inflammatory effects of statins.** Statins can inhibit the activity of NF-κB, and NF-κB is an important transcription regulatory protein in inflammatory response. In addition, statins are able to activating NOS gene transcription, and stimulate the production of NO.

Nitric oxide (NO) synthesis is catalyzed by nitric oxide synthase (NOS) with L-arginine, NADPH and molecular oxygen. It is released from the endothelium and present in a sustained form in the vascular system and also acts as an important protective factor. NO could inhibit platelet aggregation and leukocyte adhesion and infiltration, eliminate free oxygen radicals, dilate blood vessels and affect inflammatory reactions in many other ways 41. The catalysis pathway of NO mediated by NOS is a highly potentially anti-inflammatory pathway. Statins can employ a variety of mechanisms to increase the synthesis of NO through NOS. Allan et al. found that simvastatin and lovastatin exerted dose-dependent protective effects in experimental stroke models by increasing NOS levels 42. Other studies have confirmed statin can activate NOS gene transcription, extend the half-life of NOS mRNA in skin cells and inhibit mevalonate- mediated isoprenoid synthesis to increase endothelial NOS activity 43 (**Figure 4**).

## Statins are potential Anticancer Agents

Geranylgeranyl pyrophosphate and isoprenoid farnesyl pyrophosphate produced by the mevalonate pathway are able to make proteins lipidation with C-terminal motifs 44. The process of isoprenylation is essential for the function and localization of small guanosine triphosphatases (GTPases), such as Ras protein, which is a key point in cancer progression 45.

Unlimited proliferation is one of the important characteristics of cancer cells and is an important reason why cancer is difficult to cure. Ras, RhoA and other small G-proteins play critical roles in cell proliferation, metastasis and apoptosis 46, 47. As these small G-proteins require isoprenylation to be activated, their activity is fundamentally dependent on HMGCR. Statins inhibit cancer cell proliferation by disturbing the prenylation of several major small G-proteins. Different types of drugs exert various effects on the proliferation of different cancer cells. In a breast cancer mouse model, lipophilic statins, such as simvastatin, inhibit tumor growth, while the hydrophilic pravastatin did not demonstrate inhibition 48. Notably, it has been shown that the disruption of isoprenylated proteins, such as Ras and RhoA, is unlikely to mediate the anticancer activities of statins alone. This finding indicates that the loss of multiple isoprenylated proteins immediately leads to statin-induced apoptosis 49.

Signal transduction process is closely linked to cell proliferation and survival. Vosper et al. found lovastatin blocked the cell cycle in prostate cancer cells, causing cells to stay in the G1 phase, and inhibited cancer cell proliferation. Downregulation of CDKs or upregulation of cell cycle blocking factor occurred during this process. However, the addition of farnesyl pyrophosphate (FPP) or geranylgeranyl pyrophosphate (GGPP) recovered cell cycle 50. Gbelcová et al. studied the anticancer effects of different statins in pancreatic cancer and found that the effects of mevastatin on pancreatic cancer cell were superior, while in animal models, rosuvastatin was found to be better 51. These effects are associated with blocking of the Ras protein-mediated cell signal transduction.

A large number of studies have shown that cessation of the cell cycle does not depend on the lipid-lowering effects of statins, but statins inhibit cancer cell mitosis in a protease-associated manner and exert anti-proliferation effects involving the downregulation of CDK2 expression and upregulation of p21 and p27 activity 52, 53. Researchers have used proteomics methods to study the mechanisms of lovastatin resistance of breast cancer and demonstrated that the inhibition of breast cancer cells occurred through the regulation of Ras and AKT signaling molecules 54, 55.

Apoptosis is a hallmark of cancer cells and induction of cancer cell apoptosis is a hotspot for anti-cancer drug researches. It has been confirmed both *in vivo* and *in vitro* that statins can induce cancer cell apoptosis, including lovastatin and simvastatin. 56, 57. In recent years, studies have shown that statin-induced cancer cell apoptosis is involved in regulating many apoptosis signaling pathways. Jung et al. found that simvastatin activated caspase-8, caspase-3 and caspase-9 in prostate cancer cells and induced apoptosis 58. Studies from Wang et al. showed that simvastatin induced apoptosis of breast cancer cells via reducing the activity of PI3K/AKT 59. In addition, Chang et al. found simvastatin could induce apoptosis of colorectal cancer cells by activating MAPK-p53-survivin cascade 60. In short, various studies have shown that different statins may act on different tumor cells to induce apoptosis through different signaling pathways. Recently, there has also been a large number of reports indicating that statins inhibit the expansion and metastasis of cancer via the Hippo pathway. Statins regulate YAP protein entry into the nucleus by influencing the conformation of cytoskeleton 61, 62.

**Figure 5:**
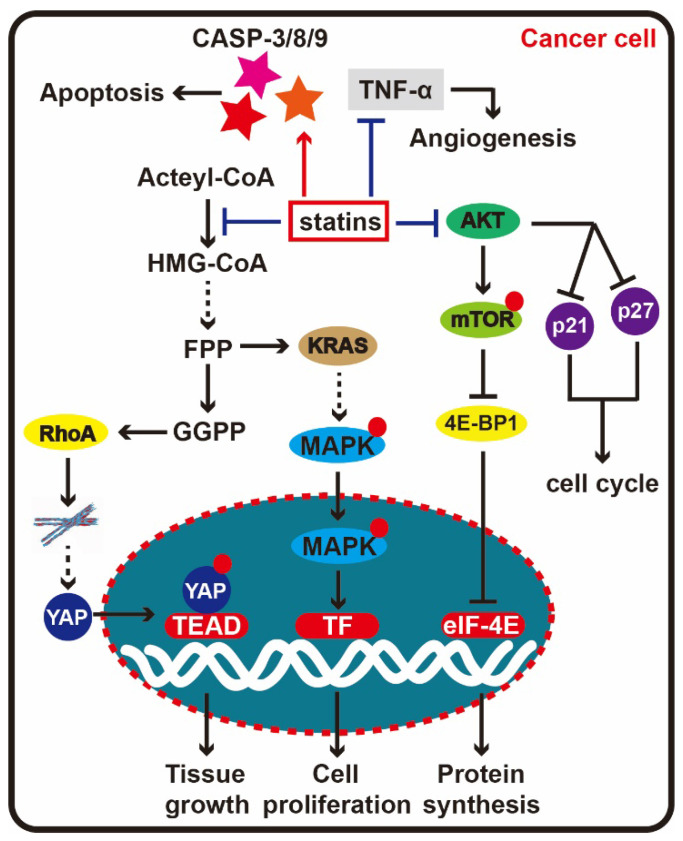
** Statin therapy for cancer.** When statins enter cells and block HMG-CoA reductase activity, the mevalonate pathway end product, RhoA is depleted. Depression of RhoA can affect the stability of the cytoskeleton, further prevent the YAP entering cell nucleus, here, YAP will drive transcription of target genes with TEAD to promote the tumor tissue growth. At the same time, KRAS is another important downstream molecule in the mevalonate pathway, which can activate the MAPK signal to accelerate cell proliferation, thus restraining KRAS will delay cancer cell proliferation. In addition to the mevalonate pathway, statins could directly depress the AKT signal, effect the synthesis and degradation p21 and p27, at last block the cell cycle progression. Besides, statin can activate caspase-8, caspase-3 and caspase-9 to induce apoptosis, and suppress angiogenesis by inhibiting TNF-α.

In many cancers, angiogenesis is induced to provide nutrients and oxygen for cancer cell growth through the transfer of vessels providing transport to other organs and parts of the body, leading to tumor metastasis. A large number of experiments have shown that statins can inhibit tumor angiogenesis. In Ras-3T3 transgenic mouse tumor models, lovastatin suppresses tumor growth and angiogenesis by inhibiting tumor necrosis factor (TNF-α) 63 (**Figure 5**). Researchers found low concentrations of cerivastatin and lovastatin enhanced endothelial cell proliferation, whereas high concentrations inhibited angiogenesis. Furthermore, these phenotypic variations are related to the prenylation of small G-proteins. Accumulating data show statins induce angiogenesis in a dose-dependent manner, that is, low concentration stimulating angiogenesis and high concentration suppressing angiogenesis 64.

## Discussion

Statins can cause many side effects while treating various diseases. The side effects of statins include liver toxicity 65, muscle toxicity 66 and neurotoxicity 67, as well as the following: effects on coenzyme Q10 and the mitochondrial membrane 68, 69; effects on isopentenyl and glycosylase, and effects on ion channel signaling 70. The dysfunction of cellular metabolism further leads to various adverse effects.

Statins are primarily metabolized by the liver and excreted by the kidneys. Therefore, if there is liver and renal failure, statins should not be administered. Alcoholics should be closely followed after using statins. Cholestasis can delay the excretion of statins, and increased plasma concentrations of statins may increase the risks of statin-induced myopathy (SIM). In addition, a history of statin drug allergy in SIM patients should also preclude the use of statins 71. Thus, we must pay more attention to their application. In the meantime, some SIM symptoms can be overcome with concomitant use of non-sterol isoprenoids.

Despite being originally developed to address the escalating problem of high cholesterol in cardiovascular disease, statins appear to have extensive untapped potential. What we need to do is to maximize the benefits of statins and try to eliminate the potential side effects. Based on clinical experience, the side effects of various statins are not identical. By comparing the toxicities of various statins in liver cancer cells, it was found that the rank order of cytotoxicity was cerivastatin > simvastatin > atorvastatin > lovastatin > pravastatin 72. Therefore, we should choose statins displaying less toxicity and fewer side effects. Moreover, Statin-induced myopathy is caused by the long-term use of higher doses, when certain drugs have been used previously (cyclosporine, erythromycin and clarithromycin), we should be fully aware of toxic side effects and other drug interactions and monitor for adverse reactions. Additionally, care should be taken in the treatment of many diseases including cancer because the underlying mechanisms are not fully clear and because the experimental results cannot necessarily be repeated. Future studies should spend more time and efforts on basic research to explore the mechanisms of statins.
